# C-853-08. Welcome to the Team: Creating a Comprehensive Orientation Program for Onboarding New Inpatient Burn APPs

**DOI:** 10.1093/jbcr/irag033.076

**Published:** 2026-04-06

**Authors:** Julia C Slater, Duncan Nickerson, Hannah Miller, Satish Ponnuru, Dhaval Bhavsar

**Affiliations:** The University of Kansas Health System Burnett Burn Center, Kansas City, KS; The University of Kansas Health System Burnett Burn Center, Kansas City, KS; The University of Kansas Health System, Kansas City, KS; The University of Kansas Health System Burnett Burn Center, Kansas City, KS; The University of Kansas Health System, Kansas City, KS

## Abstract

**Introduction:**

With resident participation in burn rotations and numbers of available burn surgeons continuing to decline, more and more burn units are augmenting their burn teams with Advanced Practice Providers (APPs), both Nurse Practitioners and Physician Assistants.

Our burn center had not hired a new APP in over a decade. With a new hire pending, straight out of their NP program, we developed an APP Orientation Program to qualify our new team member in their new role.

**Methods:**

We conducted a survey of six past and current burn APPs from three institutions and one physician assistant student that had rotated through our unit to obtain their perspectives on the list of knowledge and skill domains required for the burn APP role. We surveyed our four burn surgeons to derive the list of required domains, validating this list with a burn surgeon from another institution with significant prior involvement in APP orientation. After integrating the APP and surgeon-derived lists, we created a multi-faceted APP Orientation Program including a six month period of weekday shifts working alongside our current APPs and surgeons to introduce day to day work-flow and surgical management of burns, a first-assist course for operative training, “buddy” calls with the existing APPs to get experience with burn call overnight and on weekends, and weekly seminars on burn-specific topics delivered one-on-one by the attending surgeons on a rotating basis. A checklist was maintained to track acquisition of the required competencies.

**Results:**

After the completion of our APP Orientation Program, we surveyed our burn APPs, burn surgeons, and burn unit nursing management using a 5-point Likert scale. Most responders (88%, 7/8) strongly agreed that they were aware we had created a formal APP orientation. Of all responders, 75 % (6/8) strongly agreed that they were satisfied with the process and that the orientation successfully provided the information necessary to onboard a new APP. The rest of the responders for the previous two questions answered that they agreed with the statements. When asked if the process should be utilized for the next APP hire, 62.5% (5/8) said they strongly agreed and 37.5% (3/8) said they agreed. There were no responses that were neutral or that expressed disagreement to any of the four questions asked. Additionally, we had planned for our new APP to have six months of shift overlap with our current APPs but she was able to begin practicing independently in her role at four months.

**Conclusions:**

As more APPs are needed for coverage of burn units, a structured orientation program can make the on-boarding process smoother and more satisfying for all involved. Development of a national standard burn APP curriculum could be useful and developing an ABA certification for burn APPs similar to the FABA or CBRN designations should be considered.

**Applicability of Research to Practice:**

Directly applicable to the hiring and onboarding of new APPs in the burn unit.

**Funding for the Study:**

N/A.

